# Anthrax Lethal Toxin-Mediated Killing of Human and Murine Dendritic Cells Impairs the Adaptive Immune Response

**DOI:** 10.1371/journal.ppat.0010019

**Published:** 2005-10-28

**Authors:** Abdelkrim Alileche, Evan R Serfass, Stefan M Muehlbauer, Steven A Porcelli, Jürgen Brojatsch

**Affiliations:** Department of Microbiology and Immunology, Albert Einstein College of Medicine, Bronx, New York, United States of America; The Salk Institute for Biological Studies, United States of America

## Abstract

Many pathogens have acquired strategies to combat the immune response. *Bacillus anthracis* interferes with host defenses by releasing anthrax lethal toxin (LT), which inactivates mitogen-activated protein kinase pathways, rendering dendritic cells (DCs) and T lymphocytes nonresponsive to immune stimulation. However, these cell types are considered resistant to killing by LT. Here we show that LT kills primary human DCs in vitro, and murine DCs in vitro and in vivo. Kinetics of LT-mediated killing of murine DCs, as well as cell death pathways induced, were dependent upon genetic background: LT triggered rapid necrosis in BALB/c-derived DCs, and slow apoptosis in C57BL/6-derived DCs. This is consistent with rapid and slow killing of LT-injected BALB/c and C57BL/6 mice, respectively. We present evidence that anthrax LT impairs adaptive immunity by specifically targeting DCs. This may represent an immune-evasion strategy of the bacterium, and contribute to anthrax disease progression. We also established that genetic background determines whether apoptosis or necrosis is induced by LT. Finally, killing of C57BL/6-derived DCs by LT mirrors that of human DCs, suggesting that C57BL/6 DCs represent a better model system for human anthrax than the prototypical BALB/c macrophages.

## Synopsis

Dendritic cells (DCs) are specialized white blood cells that identify and present antigens to immune cells, T cells, in order to mount an immune response targeted against specific pathogens. DCs are critical to a host's defense against infection. Previous work has shown that the anthrax bacterium disables many immune cells, including DCs, through the action of a released toxin, lethal toxin. Here the authors show that lethal toxin efficiently kills both human and murine DCs. The means by which DCs were killed by the anthrax toxin were notably distinct and dependent on their genetic background. Human DCs, as well as those derived from the murine strain C57BL/6, died over the course of 72 h via activation of apoptosis, or programmed cell death. DCs from BALB/c mice, however, died rapidly of a necrotic cell death following toxin exposure. As human and C57BL/6 DCs share an identical response to anthrax toxin, C57BL/6 mice appear to provide an excellent model for human anthrax. The study's findings suggest that specific targeting of DCs by the anthrax toxin impairs the immune response of the infected host, and the authors believe that this strategy promotes spread of the bacterium and disease progression.

## Introduction

*Bacillus anthracis,* the causative agent of anthrax disease [1], releases lethal toxin (LT), which is sufficient to cause death in mice even in the absence of the bacterium [2]. Broad cytopathic and lethal effects associated with *B. anthracis* infection can be reproduced by LT injection of mice [3,4]. Lethal toxin consists of two components: protective antigen (PA) and lethal factor (LF). PA binds to specific cell surface receptors and mediates endocytosis of LF, a metalloprotease [5,6]. LF cleaves six of seven mitogen-activated protein kinase (MAPK) kinases (MAPKKs), thereby disrupting MAPK signaling pathways (reviewed in [7,8]). Despite ubiquitous uptake by mammalian cells, LT kills only a few cell types, including murine macrophages [3,4]. Susceptibility of murine macrophages to LT killing is strain-specific, and is controlled by a region of Chromosome 11 that encodes the kinesin-like motor protein Kif1c [9,10].

*B. anthracis* appears to combat the host immune response by inactivating MAPK pathways, which renders immune cells, including dendritic cells (DCs), nonresponsive to immune stimulation [11–13]. This may weaken the immune response by limiting the production of inflammatory cytokines [12,14–16]. However, drastic cytokine induction has been shown in several murine strains following LT injection [3,17]. These findings call into question the in vivo relevance of LT-mediated nonresponsiveness. DCs, which are considered resistant to LT-mediated cell killing [12], are key elements of the adaptive immune response, and are responsible for uptake and presentation of microbial antigens [18]. Upon maturation, DCs migrate to secondary lymphoid organs where they stimulate naïve T cells [18].

Here we report that murine and human DCs are efficiently killed by LT. The mechanism of LT-mediated DC killing is dependent on genetic background: LT triggers rapid necrotic cell death in BALB/c-derived DCs, and slow apoptosis in C57BL/6-derived DCs, as well as human DCs. Finally, we show rapid DC depletion in LT-treated BALB/c mice, suggesting direct immune impairment and in vivo relevance.

## Results

### Killing of Human DCs by Anthrax LT

**Figure 1:**
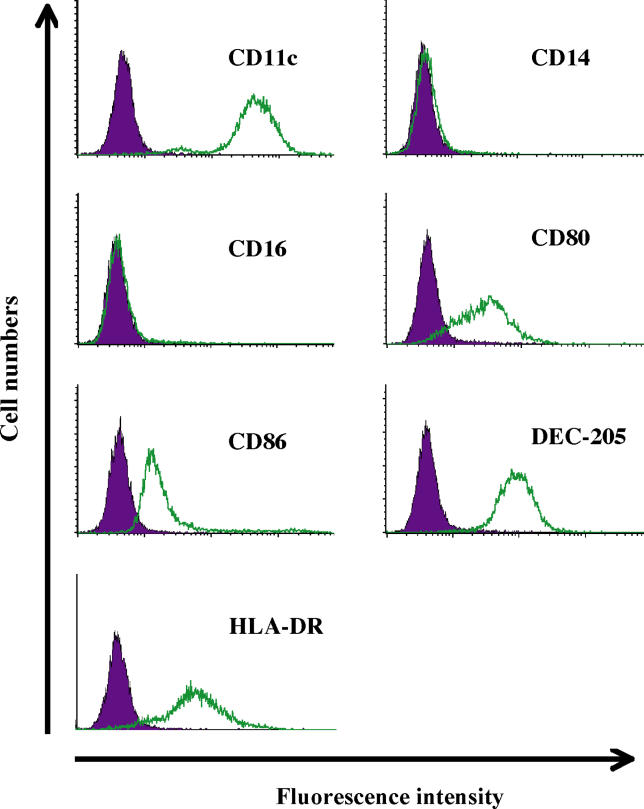
FACS Profile of Immature Human MoDCs MoDCs were derived from human peripheral blood monocytes. Expression of HLA-DR, CD11c, CD14, CD16, CD80, CD86, and DEC-205 was assessed by flow cytometry. The data were collected from two subjects and are representative of similar experiments. Filled histograms represent isotype-matched controls.

**Figure 2:**
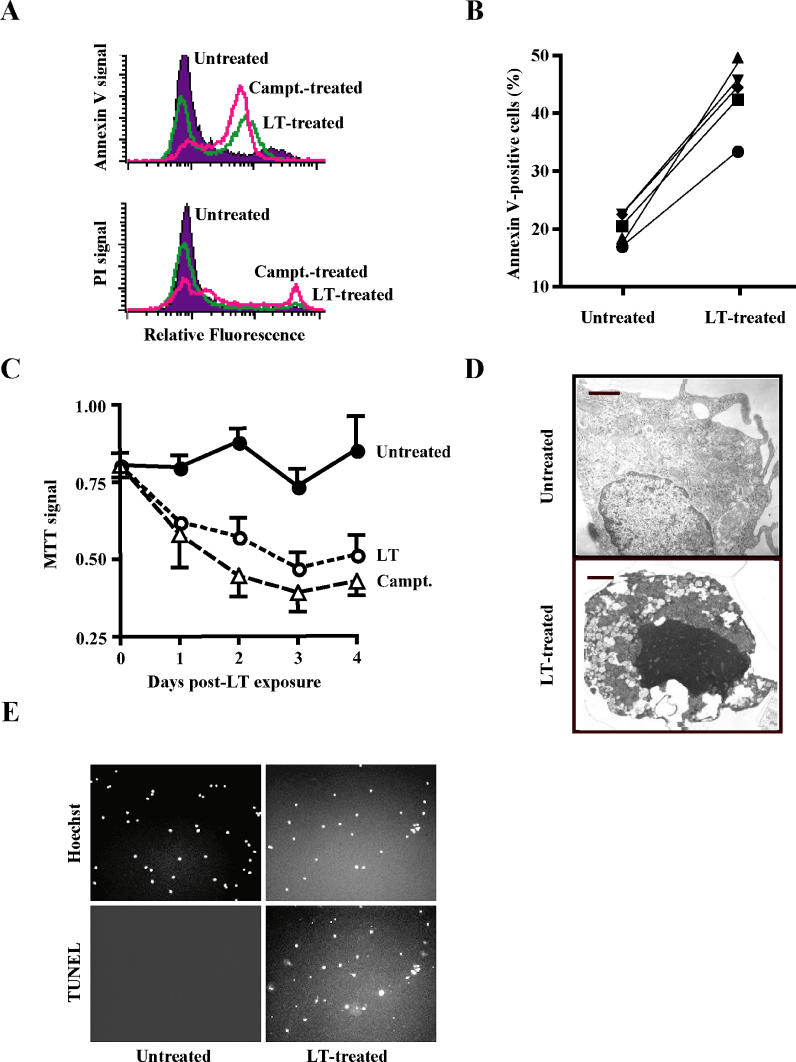
Killing of Human DCs by LT (A) MoDCs from a representative human subject were treated with LT (500 ng/ml PA and 250 ng/ml LF) or 10 μM camptothecin (Campt.) as a positive control, and annexin V/PI staining was measured by flow cytometry 48 h post-LT exposure. (B) Percentages of annexin V-positive untreated and LT-treated MoDCs from five different subjects were determined by flow cytometry 48 h post-LT exposure. (C) MTT assay of LT or camptothecin-treated human MoDCs. Mean + standard deviation from three independent experiments are shown. (D) LT-treated human MoDCs show signs of apoptotic cell death, as analyzed by electron microscopy 48 h post-LT exposure. Bars: 1 μm. (E) Human MoDCs were TUNEL-positive 48 h post-LT exposure. Untreated and LT-treated MoDCs were subjected to TUNEL and Hoechst staining.

LT-treated DCs were reported to be non-responsive to LPS stimulation, as well as impaired in their abilit7y to present antigens [12]. To determine whether the impairment of DCs could be due to cytopathic effects mediated by LT, we generated immature monocyte-derived dendritic cells (MoDCs) from human peripheral blood monocytes. MoDCs were found to be CD11c^+^, CD80^+^, CD86^+^, HLA-DR^+^, DEC-205^+^, CD14^−^, and CD16^−^ (Figure 1), consistent with markers found on immature human DCs [19]. MoDCs were challenged with a dose of LT (500 ng/ml PA and 250 ng/ml LF) cytotoxic to murine J774A.1 macrophages [20]. In contrast to previous findings, we found that human MoDCs were efficiently killed by LT [12]. LT-treated MoDCs from five different subjects showed a consistent increase in annexin V binding, while remaining propidium iodide-negative 48 h after LT challenge (Figure 2A and 2B). Induction of cytopathic effects was further supported by a drop in MTT (3-[4,5-dimethylthiazol-2-yl]-2,5-diphenyltetrazolium bromide) activity (Figure 2C). At 72 h post-LT exposure, the MTT signal declined to levels only marginally above those observed in MoDCs treated with the apoptosis inducer camptothecin (Figure 2C). No cytopathic effects were observed in MoDCs treated with PA or LF alone (unpublished data). Ultrastructural analysis of LT-treated human MoDCs revealed signs of apoptotic cell death, including chromatin condensation, pycnotic nuclei, and cytoplasmic vacuolization, along with membrane preservation (Figure 2D). Apoptosis induction was further supported by TUNEL staining of LT-treated MoDCs (Figure 2E). These results are consistent with reports of apoptosis induction following LT challenge of human endothelial cells, phorbol myristate acetate-stimulated monocytic cell lines, and lipopolysaccharide (LPS)-stimulated murine macrophages [13,21–23].

### Control of LT Killing of Murine DCs by Genetic Factors

**Figure 3:**
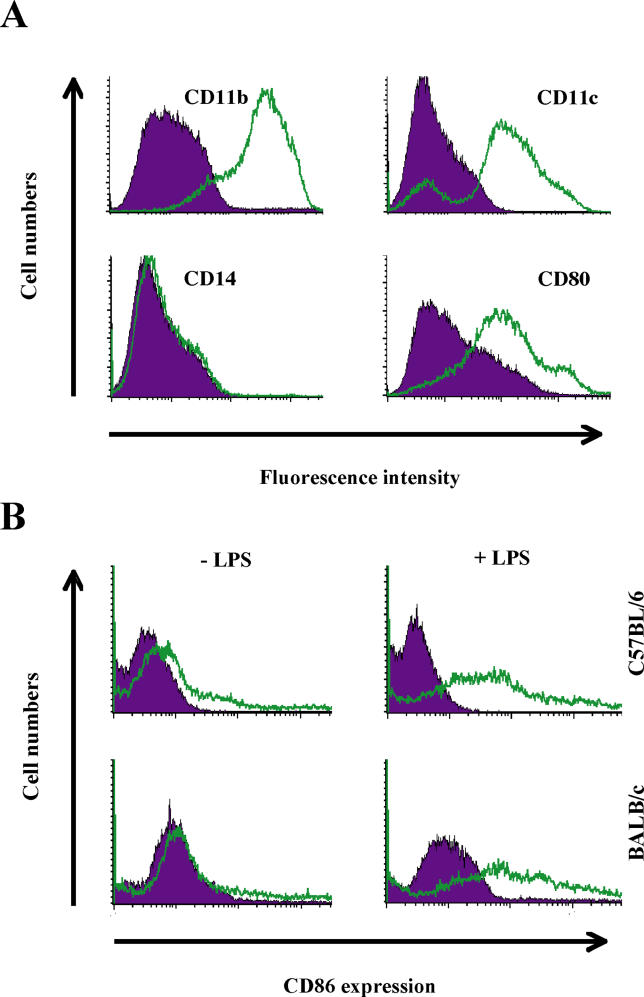
FACS Profile of BMDCs (A) BMDCs were derived from a BALB/c mouse and analyzed on day 10. Expression of CD11b, CD11c, CD14, and CD80 was assessed by flow cytometry. The data are representative of four similar experiments in BALB/c and C57BL/6 mice. (B) BALB/c and C57BL/6-derived BMDCs were stimulated by LPS for 18 h, and CD86 expression was measured by flow cytometry. Filled histograms represent isotype-matched controls.

To test whether murine DCs were also susceptible to LT killing, we generated immature bone marrow-derived dendritic cells (BMDCs) from BALB/c and C57BL/6 mice [24]. BMDCs were CD11b^+^, CD11c^+^ (over 80%), and CD14^−^, and they expressed low levels of CD80 and CD86 (Figure 3A and 3B), consistent with surface markers found on in vitro-generated immature murine DCs [25,26]. To ensure that we were working with immature DCs, we treated these DCs for 18 h with LPS, and confirmed maturation by measuring surface levels of CD86. As expected, LPS exposure for 18 h generated a significant up-regulation in CD86 surface expression levels (Figure 3B). Exposure of DCs to PA or LF (1 μg/ml) alone did not result in CD86 up-regulation (unpublished data).

**Figure 4:**
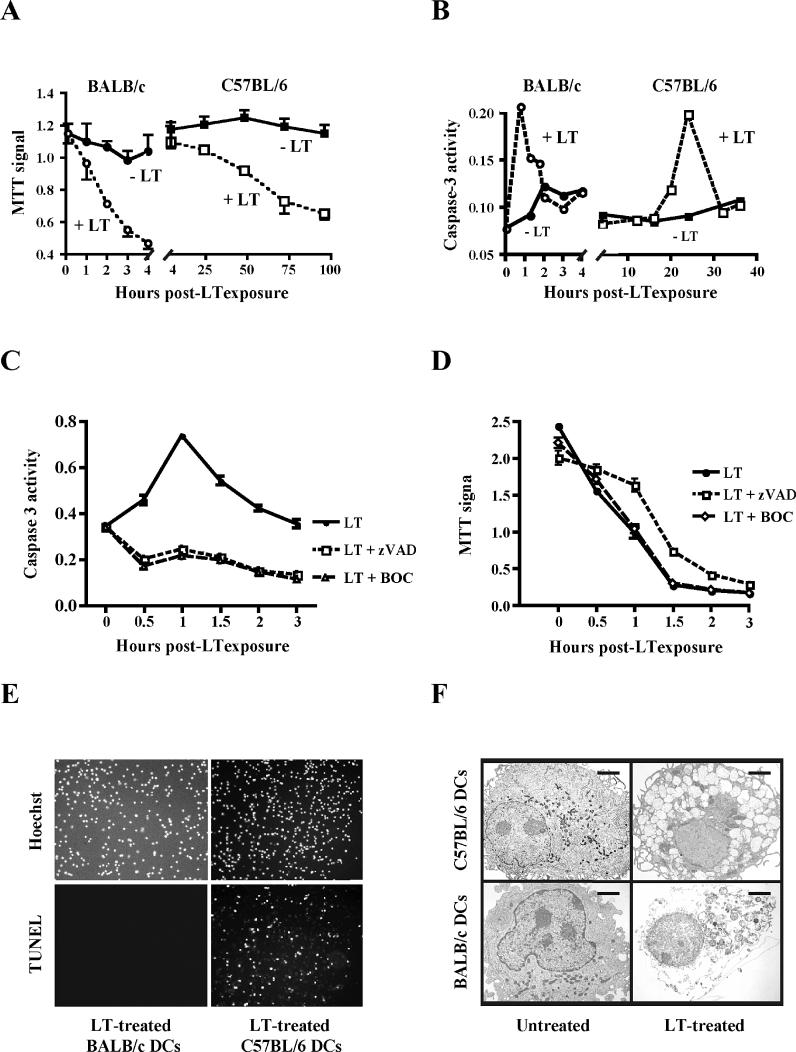
BALB/c- and C57BL/6-Derived DCs Differ in Their Response to LT (A) BALB/c and C57BL/6-derived BMDCs were treated with LT (500 ng/ml PA and 250 ng/ml LF), and cell survival was determined by MTT assay. (B) Caspase-3 activation of LT-treated BALB/c and C57BL/6 DCs as determined by a colorimetric caspase-3 cleavage assay. A representative experiment is shown. (C) The caspase inhibitors Z-VAD-FMK (10 μg/ml) and BOC-D-FMK (40 μg/ml) prevent caspase-3 activation in LT-treated BALB/c BMDCs. (D) The caspase inhibitors Z-VAD-FMK (10 μg/ml) and BOC-D-FMK (40 μg/ml) do not prevent LT killing of BALB/c BMDCs as determined by MTT assay. (E) C57BL6, but not BALB/c DCs, were TUNEL-positive post-LT exposure. BALB/c and C57BL/6-derived BMDCs were treated with LT (500 ng/ml PA and 250 ng/ml LF), and were stained using a TUNEL reaction and a Hoechst counterstain. (F) BALB/c and C57BL/6-derived BMDCs were analyzed by electron microscopy 4 and 48 h post-LT exposure, respectively. Bars, 1 μm.

BMDCs from BALB/c and C57BL/6 mice were exposed to LT, and cell viability was determined at several time points post-LT exposure (Figure 4). The MTT signal dropped significantly in BALB/c-derived BMDCs within 2–4 h of LT challenge. Cell viability fell in C57BL/6-derived BMDCs as well, but only after 2–3 d of LT exposure (Figure 4A). Accordingly, BALB/c and C57BL/6-derived BMDCs underwent morphological changes 2 h and 2 d post-LT exposure, respectively, as determined by phase contrast microscopy (unpublished data). In addition to immature BMDCs, we isolated splenic DCs with a mature phenotype from BALB/c mice using magnetic beads. These splenic DCs died within 4 h of LT exposure as determined by morphological changes and MTT assays (unpublished data), suggesting that LT susceptibility of murine DCs is independent of their maturation state.

Our findings are consistent with the in vitro sensitivity of murine macrophages to LT killing, which is also dependent on genetic background [3,4,27]. BALB/c-derived macrophages are efficiently killed by LT with rapid kinetics, while C57BL/6-derived macrophages are resistant to rapid LT killing [13]. Similarly, in vivo studies have shown rapid disease progression in LT-treated BALB/c mice, and a delayed onset of lethal effects in LT-treated C57BL/6 mice [3,4].

As apoptotic pathways were activated in LT-treated human DCs (see Figure 2D and 2E), we tested for signs of apoptosis in murine BMDCs following LT exposure. C57BL/6 BMDCs showed clear signs of apoptosis, as indicated by TUNEL staining and nuclear condensation (Figure 4E and 4F). In contrast, no chromatin condensation, TUNEL staining, or DNA fragmentation was detected in LT-treated BALB/c BMDCs (Figure 4E and 4F), which is consistent with rapid necrotic cell death, as has been observed in LT-treated BALB/c-derived macrophages [13,23]. Strikingly, LT exposure triggered caspase-3 activation in murine BMDCs derived from both BALB/c and C57BL/6 mice. Consistent with the cell killing kinetics described above, caspase-3 activation occurred rapidly in BALB/c-derived BMDCs (1 h post-LT exposure), and slowly in C57BL/6-derived BMDCs (24 h post-LT exposure) (Figure 4B and 4C). We used the pan-caspase inhibitors Z-VAD-FMK and BOC-D-FMK to block caspase activation. Both caspase inhibitors blocked caspase-3 activation (Figure 4C), but failed to block LT killing of BALB/c-derived DCs (Figure 4D), indicating that caspase activation was not required for induction of cytopathic effects in these cells. Experiments using caspase inhibitors were limited to BALB/c DCs, as both Z-VAD-FMK and BOC-D-FMK showed high toxicity in murine DCs when used for more than 12 h.

**Figure 5:**
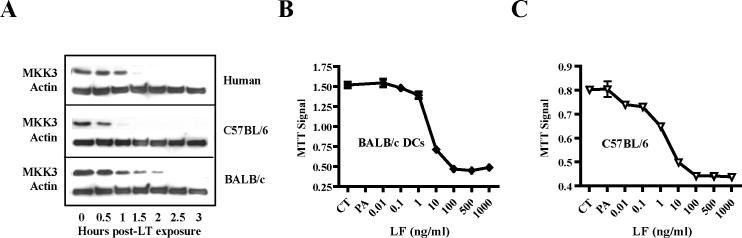
MAPKK Cleavage Kinetics and LT Susceptibility of BALB/c- and C57BL/6-Derived BMDCs (A) MAPKK-3 cleavage occurs at similar rates in murine and human DCs treated with LT (500 ng/ml PA and 250 ng/ml LF), as determined by Western blot analysis using anti-MKK3 and anti-actin (control) antibodies. (B and C) Relative LT susceptibility of BALB/c- (B) and C57BL/6-derived (C) BMDCs. BALB/c and C57BL/6 BMDCs were subjected to PA (500 ng/ml) and varying concentrations of LF. After 4 h (BALB/c DCs) and 72 h (C57BL/6 DCs), cell viability was determined by MTT assays. Representative experiments are shown.

These results revealed that the genetic background of murine DCs determines LT killing kinetics and cell death pathway activation. The difference in the LT killing kinetics between BALB/c and C57BL/6 BMDCs or human DCs was not caused by a discrepancy in LT uptake, MAPKK-3 cleavage, or LT sensitivity. MAPKK-3 cleavage, the earliest indicator of LF action, occurred in BALB/c BMDCs, C57BL/6 BMDCs, and human MoDCs at similar time points, as determined by Western blotting (Figure 5A). The small divergences in MAPKK-3 cleavage did not correlate with differences in LT killing kinetics, suggesting that these kinetics were not determined by the rate of MAPKK-3 cleavage, and were presumably caused by downstream events.

To determine the LT sensitivity of BALB/c and C57BL/6-derived BMDCs to LT killing, we treated these cells with increasing LF concentrations, while keeping PA constant at 500 ng/ml. Both BALB/c and C57BL/6-derived BMDCs exhibited similar susceptibility to LT, with the main increase in LT killing occurring between 1 and 10 ng/ml of LF (Figure 5B and 5C). Taken together, our results indicate that the difference in LT killing cannot be explained by divergences in MAPKK cleavage or LT sensitivity.

### Control of LT Killing of Murine DCs by Proteasomal Activity

**Figure 6:**
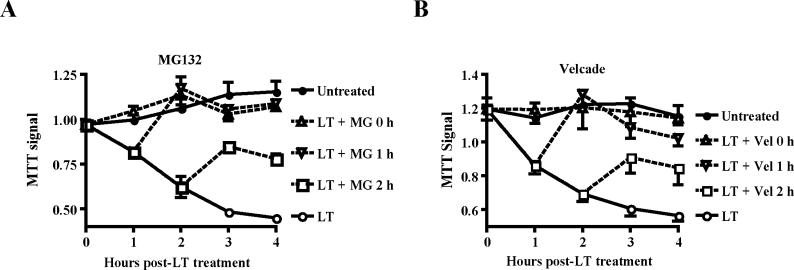
Proteasome Inhibitors Block LT-Mediated Killing of BALB/c-Derived DCs Proteasome inhibitors MG132 (10 μM) (A), or Velcade (0.1 μM) (B) were added either simultaneously with LT or 1 or 2 h post-LT exposure, and cell viability was determined by MTT assay. Mean + standard deviation of four independent experiments are shown.

LT killing of BALB/c-derived murine macrophages is controlled by the proteasome system [20,28]. To determine whether BALB/c-derived DCs also require proteasomal activity for LT killing, we added the proteasome inhibitors MG132 or Velcade (bortezomib) to BALB/c-derived BMDCs at different time points following exposure to LT. Both proteasome inhibitors completely blocked LT killing when added simultaneously with LT to BALB/c-derived DCs (Figure 6). Strikingly, the MTT signal recovered completely from an initial drop when proteasome inhibitors were added to BALB/c BMDCs up to 1 h post-LT exposure (Figure 6). Partial recovery was obtained when the proteasome inhibitors were added 2 h post-LT treatment (Figure 6). These results suggested that proteasomal degradation of protective factors controls a late step in LT killing of BALB/c DCs. Experiments using proteasome inhibitors were limited to BALB/c DCs, as both MG132 and Velcade were highly toxic to DCs when applied for more than 8 h. It is conceivable that slow LT killing of C57BL/6-derived cells is due to increased levels or stability of putative protective factors. Proteasomes may also control LT-induced morbidity and mortality in LT-treated mice.

### In Vivo Depletion of DCs and Loss of T Cell Activating Function Following LT Exposure

**Figure 7:**
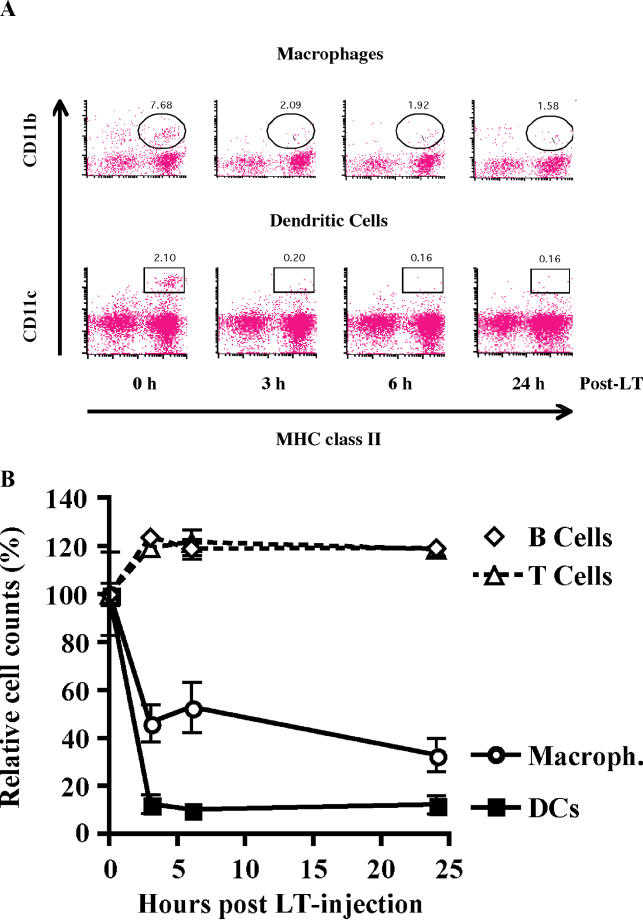
In Vivo Depletion of DCs and Loss of T Cell Activating Function Following LT Exposure (A) Murine DCs and macrophages are killed by LT in vivo. Ten-week-old BALB/c mice were injected intraperitoneally with LT, and the percentage of DCs and macrophages in the spleen were determined by flow cytometry. A representative experiment is shown. (B) Specific depletion of DCs and macrophages in LT-treated BALB/c mice injected intraperitoneally with either PBS or LT (200 μg PA and 200 μg LF). Levels of splenic DCs (CD11c^+^, MHC class II^+^), macrophages (CD11b^+^, MHC class II^+^), and circulating B cells (B220^+^) and T cells (CD3^+^) were determined by flow cytometry. Percent changes are shown by number of cells derived from LT-treated BALB/c mice (three mice per time point), relative to PBS-treated control mice (three mice per control experiment).

To determine whether murine DCs were also killed by LT in vivo, we injected 10-wk-old female BALB/c mice intraperitoneally with LT (200 μg PA and 200 μg LF), and analyzed bulk splenocytes from these mice using flow cytometry. We selected BALB/c mice, the commonly recognized prototype strain for anthrax research, for our in vivo experiments. Consistent with our in vitro data, levels of splenic DCs dropped rapidly in LT-treated BALB/c mice (Figure 7). Levels of DCs declined to 10% of those found in untreated control mice 3 h after LT challenge, and remained at this level for at least 24 h. Levels of splenic macrophages were also reduced, reaching 20% of control levels 24 h post-LT challenge (Figure 7B). As expected, levels of B and T cells, which are resistant to LT killing, did not drop after LT exposure (Figure 7B). These results showed that LT killing of DCs was not restricted to the cell culture system, as it also occurs in vivo. LT-treated DCs have been described as impaired in their abilities to respond to cytokines and to present antigens [12]. Our findings indicate that significant impairment of the immune response in LT-treated mice is caused by rapid DC depletion.

## Discussion

Here we show for the first time, to our knowledge, that anthrax LT is highly toxic to human, BALB/c, and C57BL/6 DCs, even at very low concentrations. In a previous report, Tournier et al. [29] showed that infection of C57BL/6 and BALB/c DCs by anthrax spores disrupts the cells' ability to produce cytokines. The authors also reported a loss of viability in BALB/c DCs treated with a combination of *B. anthracis* spores and purified LT. Our results show that purified LT alone was sufficient to reproduce these observations.

Our findings on DC killing challenge an earlier report [12], which described DC impairment without cell killing. Agrawal et al. [12] specifically reported no cytopathic effects in LT-treated C57BL/6-derived DCs, as measured by Trypan blue exclusion. This is not surprising, as LT-mediated killing of C57BL/6 BMDCs occurred without significant membrane perturbation. Additionally, they reported no caspase-3 activation in C57BL/6 DCs at 6 and 48 h post-LT exposure. We found that a window of caspase-3 activation occurred between 18 and 32 h (see Figure 4B). At the time points reported in their study, we also found no increase in active caspase-3.

We show that genetic background determines the type of cell killing induced by LT: LT triggered rapid necrosis in BALB/c-derived DCs and slow apoptosis in C57BL/6-derived DCs. A genetic polymorphism containing a putative susceptibility factor at the *Kif1c* locus on Chromosome 11 has been identified that correlates with the sensitivity of macrophages to LT killing [9,30]. We believe that the strain-specific cell death pathways we report are also controlled by this genetic locus. It remains to be shown how genetic factors determine whether apoptotic or necrotic pathways are induced by LT.

DC killing precedes lethal effects in LT-treated BALB/c and C57BL/6 mice by approximately 24 h, and therefore constitutes an early event in anthrax pathogenesis [3]. The reason for selective LT targeting of antigen presenting cells, including macrophages and DCs, is unknown. Murine C57BL/6 DCs and human DCs share slow killing kinetics and induction of apoptosis following LT exposure. Therefore, C57BL/6-derived cells appear to be a better model system for human anthrax than the prototypical BALB/c-derived macrophages, which rapidly undergo necrotic cell death after LT challenge. Induction of distinct cell death pathways suggests that LT-mediated cell killing is controlled by strain-specific regulators and inhibitors. Extensive investigation has led to the discovery of multiple potent apoptosis inhibitors, which might block LT-mediated apoptosis, and possibly anthrax disease progression, in C57BL/6 mice and human subjects. Additionally, these findings might be important for therapeutic applications. It is conceivable that agents that block LT killing are strain-specific, and that drugs blocking LT-mediated killing of BALB/c-derived cells might be inefficient in targeting LT killing of C57BL/6-derived and human cells and vice versa. Based on our findings, it is now possible to differentiate, in LT-treated C57BL/6 mice, cells that are directly killed by LT, presumably by apoptosis induction, from cells killed by indirect means via induction of necrotic pathways.

*B. anthracis* infections appear to challenge the immune system in two phases. During the early phase of infection, subtoxic concentrations of LT disrupt MAPK signaling pathways, rendering immune cells nonresponsive to cytokine and immune stimulation [12,13]. We present evidence that this phase is followed by LT killing of DCs, which presumably occurs when the concentration of LT reaches cytotoxic levels. This likely causes additional impairment of adaptive immunity, and represents an immune-evasion strategy of the bacterium. DCs as well as macrophages are primary entry sites of *B. anthracis* [29,31,32]*,* and early replication of the bacterium occurs exclusively within these cells. The slow LT killing kinetics found in C57BL/6 and human DCs may promote bacterial proliferation.

Specific targeting of DCs by LT should prevent stimulation of the adaptive immune response, rendering hosts highly susceptible to other microbial pathogens. Depletion of DCs also plays a critical role in human and murine sepsis [33]. Additionally, it is possible that LT killing of DCs could be exploited in therapeutic applications of the toxin. LT's potential for medical applications is not novel; sublethal LT treatment was employed in nude mice containing human melanoma xenograft tumors, resulting in tumor regression without any cytotoxic side effects [34]. Due to the high specificity and sensitivity of DCs to LT, exposure of DCs to specific toxin concentrations could represent a useful approach to transiently suppress the immune response, if cytotoxic effects of LT on other cell types could be effectively controlled or eliminated.

## Materials and Methods

### Cell culture and materials.

C57BL/6 and BALB/c mice were obtained from Jackson Laboratories (Bar Harbor, Maine, United States). Human MoDCs were cultured in RPMI-1640 containing 2 mM L-glutamine and supplemented with 1% nonessential amino acids, 10 mM Hepes, 1% penicillin-streptomycin, and 10% FCS (R10 medium). The pan-caspase inhibitors Z-VAD-FMK and BOC-D-FMK (Calbiochem, San Diego, California, United States) and the proteasome inhibitor MG132 (Oncogene, Boston, Massachusetts, United States) were reconstituted in DMSO and used at a concentration of 10 μg/ml, except for BOC-D-FMK, which was used at 40 μg/ml. The proteasome inhibitor Velcade (bortezomib) was generously provided by Dr. Roman Perez-Soler (Albert Einstein College of Medicine). Recombinant anthrax LF was obtained from List Biological Laboratories (Campbell, California, United States). PA was generously provided by Dr. Steven Leppla (National Institutes of Health). PA and LF were reconstituted in water at 500 ng/ml and 250 ng/ml, respectively [35]. Recombinant LF and PA were produced in *B. anthracis* and were free of LPS contamination as indicated by the manufacturer, and by a lack of CD86 up-regulation on immature cells, as measured by flow cytometry.

### Generation of human DCs from peripheral blood.

Immature DCs were prepared from human PBMCs as previously described [36]. Briefly, PBMCs from buffy coats were isolated by centrifugation on Ficoll-Paque Plus (Amersham Biosciences, Little Chalfont, United Kingdom) at 600 *g* for 20 min. After washing three times with PBS to remove platelets, PBMCs were resuspended in 60 ml of R10 medium (containing 10% FCS and 6.6 ng/ml human GM-CSF [Leukine; Berlex, Montville, New Jersey, United States]). After 30 min at 37 °C, the medium was removed and adherent cells were cultured in R10 medium containing human GM-CSF at 6.6 ng/ml and human IL-4 (PeproTech, Rocky Hill, New Jersey, United States) at 10 ng/ml for 3–4 d, to produce immature DCs. The identity of these cells was confirmed by flow cytometry using anti-CD11c, CD14, CD16, CD80, CD86, HLA-DR, and DEC-205 antibodies.

### Generation of murine bone marrow-derived and splenic DCs.

BMDCs were prepared as previously described [24]. 10 ml syringes fitted with 25 gauge needles and filled with mR10 medium (RPMI-1640, 10% FCS, 10 mM Hepes, 1% penicillin-streptomycin, and 55 μM beta-mercaptoethanol) were used to flush bone marrow cells from the femurs and tibias of C57BL/6 and BALB/c mice. Red blood cells were lysed using RBC lysing buffer (Sigma, St. Louis, Missouri, United States), and the remaining cells were washed with PBS. Cells were plated in bacterial plates in mR10 medium containing 20 ng/ml murine GM-CSF (PeproTech). On days 3, 6, and 8, cells were resuspended in fresh mR10 containing 20 ng/ml murine GM-CSF. On day 10, nonadherent cells were resuspended in mR10 containing 5 ng/ml murine GM-CSF, for maintenance of differentiated DC populations. The identity of cells was assayed by flow cytometry using anti-CD11b, CD11c, CD14, CD80, CD86, and MHC class II antibodies. Splenic DCs were isolated using anti-CD11c magnetic beads according to the manufacturer's instructions (Miltenyi Biotec, Bergisch Gladbach, Germany). Spleens from BALB/c mice were treated with collagenase D, cells were incubated with anti-CD11c microbeads, and splenic DCs were enriched using MS separator columns.

### Cell death and viability assays.

Cell viability was measured by analysis of MTT cleavage to formazan by succinate dehydrogenases in living cells [37]. For the colorimetric MTT assay, cells were exposed to LT (500 ng/ml PA and 250 ng/ml LF), and the MTT solution (5 mg/ml MTT in PBS) was added directly to wells and incubated at 37 °C for 4 h. The dye was solubilized with acidic isopropanol (25 mM HCl and 0.5% SDS in isopropanol), and the absorbance was measured at 570 nm.

For analysis of caspase activation, we used a colorimetric caspase-3 assay, performed as recommended by the manufacturer (R&D Systems, Minneapolis, Minnesota, United States). LT-treated cells were cultured in 24-well plates and lysed in the wells after removing the culture medium. Cleavage of the substrate was measured on an LS-50 fluorescence spectrometer (Perkin Elmer, Wellesley, California, United States). TUNEL assays were performed as described previously [38].

### Western blotting.

Cells were cultured in 24-well plates and treated with LT. Western blotting was performed as described previously [39]. In brief, cells were lysed in the wells after removing culture medium. For lysis, the caspase-3 lysis buffer (R&D Systems) was supplemented with a cocktail of protease inhibitors (Roche, Basel, Switzerland). Cellular lysates were centrifuged, and supernatants were mixed with SDS sample buffer and denatured at 100 °C for 3 min. Size fractionation was performed on 50 μg of protein from each sample on BioRad SDS-Tris HCl polyacrylamide gels (BioRad, Hercules, California, United States), and transferred to PVDF membranes (Amersham). Membranes were probed with anti-MEK-3 polyclonal antibody (Santa Cruz Biotechnology, Santa Cruz, California, United States; #Sc-960) and anti-actin monoclonal antibody (Sigma; #Ac-40). Polyclonal HRP-conjugated anti-rabbit antibodies were used as secondary antibodies (Santa Cruz Biotechnology; #Sc-2313), and blots were developed using ECL Plus solution (Amersham).

### Transmission electron microscopy.

Samples were fixed with 2.5% glutaraldehyde in 0.1 M sodium cacodylate buffer, and postfixed with 1% osmium tetroxide, followed by addition of 1% uranyl acetate. After dehydration through a graded series of ethanol washes, samples were embedded in LX112 resin (LADD Research Industries, Williston, Vermont, United States). Ultrathin sections were cut on a Reichert Ultracut UCT, stained with uranyl acetate followed by lead citrate, and viewed on a JEOL 1200EX transmission electron microscope at 80 kV.

### Flow cytometry.

Human DCs and murine BMDCs were analyzed by flow cytometry using FACScan and FACScalibur flow cytometers (BD Biosciences, Palo Alto, California, United States). For each staining condition,10^6^ cells were washed once with buffer A (PBS containing 1% BSA and 0.05% NaN_3_). After blocking for 10 min with heat-inactivated fetal calf serum, primary antibody was added for 30 min on ice. Subsequently, cells were spun down, washed in buffer A and stained with secondary antibodies when necessary. The following antibodies were purchased from BD Biosciences: FITC-conjugated anti-CD14 (clone M5E2), anti-CD16 (clone 368), and anti-CD80 (clone L307.4); and PE-conjugated CD11c (clone B-Ly6) and anti-CD86 (clone 2331). The following antibodies were produced from hybridoma lines that were cultivated in our laboratory: anti-HLA-DR (clone L243; obtained from ATCC, Manassas, Virginia, United States), anti-DEC-205 (clone Mg38; obtained from Dr. R. Steinmann, Rockefeller University), and anti-CD58 (clone TS2/9; obtained from ATCC). FITC-conjugated goat F(ab′)_2_ anti-mouse IgG + IgM, fluorescein conjugate (Biosource, Camarillo, California, United States) was used as a secondary antibody when necessary. In all cases, antibody staining was compared to the appropriate isotype control.

### In vivo assay.

Ten-week-old female BALB/c mice were injected intraperitoneally with LT [3,4]. Mice were sacrificed 0, 3, 6, and 24 h after LT injection. Spleens were harvested and treated with 400 U/ml collagenase D (Roche) to release DCs. Levels of splenic DCs (CD11c^+^, MHC class II^+^), macrophages (CD11b^+^, MHC class II^+^), and circulating B cells (B220^+^) and T cells (CD3^+^) were determined by flow cytometry using fluorescently labeled antibodies to surface markers.

## Abbreviations

BMDC: bone marrow-derived dendritic cell
DC: dendritic cell
LF: lethal factor
LPS: lipopolysaccharide
LT: lethal toxin
MAPK: mitogen-activated protein kinase
MoDC: monocyte-derived dendritic cell
PA: protective antigen
